# Structural Characterization of Linker Shielding in ADC Site-Specific Conjugates

**DOI:** 10.3390/pharmaceutics17121568

**Published:** 2025-12-05

**Authors:** Maru Jaime-Garza, Andrew Waight, Manish Hudlikar, Michael J. Eddins, Elnaz S. Rasti, Jan Paulo T. Zaragoza, Laurence Fayadat-Dilman, Jill E. Chrencik, Sandra B. Gabelli, Yun-Ting Chen, Cameron L. Noland

**Affiliations:** 1 Discovery Chemistry, Merck & Co., Inc., 213 East Grand Ave., South San Francisco, CA 94080, USA; maru.jaimegarza@merck.com (M.J.-G.); manish.hudlikar@merck.com (M.H.);; 2 Discovery Biologics, Merck & Co., Inc., 213 East Grand Ave., South San Francisco, CA 94080, USA; 3 Discovery Chemistry, Merck & Co., Inc., 770 Sumneytown Pike, West Point, PA 19486, USA; 4 Discovery Chemistry, Merck & Co., Inc., 126 E. Lincoln Ave., Rahway, NJ 07065, USA

**Keywords:** antibody–drug conjugates (ADCs), site-specific conjugation, linker–payload design, trastuzumab, X-ray crystallography, hydrophobicity, Fc engineering, linker stability, ADC structure

## Abstract

**Background/Objectives**: Antibody–Drug Conjugates (ADCs) have rapidly evolved from early, rudimentary conjugates to highly targeted and precisely engineered molecules. Despite notable clinical successes, ADCs continue to face significant challenges, including aggregation and high hydrophobicity driven by high drug-to-antibody ratios (DARs), premature payload release, dose-limiting toxicities, and suboptimal pharmacokinetics. While site-specific linker–payload conjugation has improved ADC homogeneity and stability, the structural basis of antibody–linker interactions at specific sites remains underexplored. **Methods**: In this work, we present the crystal structures of trastuzumab Fab and Fc domains site-specifically conjugated with a cleavable linker–payload. **Results**: Our findings suggest that pockets within both Fab and Fc regions may interact with and shield the linker portion of the conjugate. **Conclusions**: These insights highlight the previously underappreciated potential of structure-based design to drive the optimization of ADC linker chemistry and facilitate the co-design of bespoke linker–payloads tailored to individual antibody conjugation sites.

## 1. Introduction

Over the past several decades, research on Antibody–Drug Conjugates (ADCs) has surged, leading to an increase in FDA approvals and the rapid evolution of the ADC modality [1,2,3]. ADCs uniquely combine the target-specificity and extended half-life of antibodies with the potent cytotoxicity of small-molecule chemotherapeutic payloads [4]. Despite significant clinical advancements, ADCs continue to face substantial challenges on the path to clinic [5,6,7]. Current engineering efforts focus on optimizing target selection, antibody design, and the chemistry of both linkers and payloads [4].

Early ADCs, although highly potent, relied on non-specific conjugation to antibody cysteines or lysines, resulting in heterogeneous mixtures with variable Drug-to-Antibody Ratios (DARs) within therapeutic batches [8,9,10]. This heterogeneity complicated dosing, as different DAR species exhibited distinct potencies and pharmacokinetics, underscoring the need for more uniform ADC preparations [8]. The advent of site-specific conjugation—through engineered cysteines, lysines, or unnatural amino acids—has enabled the production of more homogeneous ADCs with well-defined properties, facilitating systematic optimization [11,12,13,14].

Importantly, early studies revealed that the site of conjugation profoundly influences ADC behavior [15,16,17]. Often conjugated to engineered cysteines through Michael addition of a maleimide handle [11], placement of antibody payloads at different positions affects pharmacokinetics and therapeutic efficacy. Some conjugation sites are prone to premature payload release [18,19,20,21], while others exhibit faster maleimide hydrolysis [22], leading to irreversible conjugation [23]. Moreover, increasing DAR does not necessarily increase toxicity but often accelerates metabolic clearance [8]. Interestingly, conjugation at solvent-exposed sites leads to increased hydrophobicity and potentially promotes premature payload release [16,24].

Central to ADC design is the linker, which must remain stable in circulation while enabling efficient payload release within the target cell [3,20,25,26]. The development of cleavable and non-cleavable linkers has allowed more precise control over payload release [5]. Cleavable linkers, despite increased systemic toxicity, have been favored clinically due to their enhanced therapeutic effects [6,26]. To date, 12 of the 13 ADCs approved for the clinic use cleavable linkers. While non-cleavable linkers exhibit increased stability in circulation, it is hypothesized that their charged payloads remain trapped in cells, limiting the bystander effect, a phenomenon where ADC payloads permeate and kill neighboring cells that may not express the ADC antigen. Balancing the stability of non-cleavable linkers with the bystander killing effect of cleavable linkers has inspired innovative ADC designs, such as non-cleavable payloads that lead to the bystander effect [27,28]. As protein engineers and chemists continue to develop improved ADCs, structural insights become valuable tools for rational design.

Despite advances in computational modeling, there remains a notable gap in experimentally determined 3D structures for ADCs, limiting our understanding of their molecular interactions and design principles. In this study, we address this gap by presenting high-resolution X-ray crystallographic structures of site-specific Fab and Fc fragment conjugates. Given the foundational research on its site-specific conjugates, we use as a model ADC the well-studied Her2 receptor-targeting antibody trastuzumab conjugated with a cleavable Ala-Ala p-aminobenzyloxycarbonyl (PABC) linker to the potent antimitotic payload monomethyl auristatin E (MMAE) [11]. By directly visualizing linker–antibody interactions at defined conjugation sites, our findings reveal how structural features may contribute to reduced ADC hydrophobicity and enhanced linker stability. These insights establish a foundation for structure-guided optimization of ADCs, enabling the rational design of linker–payloads tailored to specific antibody sites and advancing the next generation of targeted therapeutics.

## 2. Materials and Methods

### 2.1. Molecular Dynamics Surface Area Calculations

Molecular dynamics simulations were calculated using PDB structures 5GGS (Fab region) and 3AVE (Fc region) as templates and were first prepared using Maestro Bioluminate 5.0.128 (Schrodinger) with histidine residues converted to HIE protonation state. Trajectories were performed using GROMACS 2024.4 with the AMBER14SB force field, where the protein was solvated in a cubic box (minimum 2.0 nm protein-edge distance) with SPC/E water molecules and neutralized with Na^+^/Cl^−^ ions. The system underwent energy minimization followed by NVT and NPT equilibration before a production simulation of 100 ns for the Fab structure and 200 ns for the Fc structure. Trajectory analysis was conducted using MDTraj (version 1.9.9) to calculate residue-level solvent-accessible surface area (SASA) via the Shrake-Rupley algorithm and Cα root mean square fluctuation (RMSF) for flexibility assessment. Residues were renumbered according to the Kabat scheme using ANARCI for both heavy chain variable (VH, Chain A) and light chain variable (VL, Chain B) regions, with data visualization performed using Plotly (version 5.24.1) to display time-averaged SASA and RMSF values. UCSF ChimeraX’s (version 1.9) “define attribute” function was used to display SASA values onto trastuzumab model.

### 2.2. Cloning, Protein Expression, and Purification

The DNA sequences used for structural characterization are as shown in Table 1 and were cloned into pTT5 vector backbones by Genscript:

Fab and Fc proteins were expressed using ThermoFisher’s ExpiCHO expression system following the manufacturer’s protocol (Waltham, MA, USA). In the case of the Fab constructs, heavy and light chain pTT5 plasmids were co-transfected at a 1:1 ratio. Expression was allowed to proceed for 7 days before protein purification. Cell pellets were spun down at 3000× *g* for 20 min, and the expression supernatant was filtered using a 0.22 μm filter. The filtered supernatant was then run through 5 mL HiTrap MabSelect columns (MabSelect PrismA for Fab purification or MabSelect SuRe for Fc purification; Cytiva, Marlborough, MA, USA) twice to maximize protein yield. The protein was then eluted using 100 mM Glycine buffer at pH 3.5. Upon elution the protein was immediately diluted with PBS and concentrated to a volume smaller than one milliliter for size exclusion chromatography on a HiLoad 16/600 Superdex 75 column (Cytiva) that had been pre-equilibrated in reduction buffer (100 mM Tris pH 8.0, 10 mM EDTA, PBS).

### 2.3. Linker–Payload Conjugation

IgG1 Fc and trastuzumab Fab were decapped in reduction buffer (100 mM Tris pH 8.0, 10 mM EDTA, PBS). A fresh stock of 10 mM 4,4′-(phenylphosphinidene)bis(benzenesulfonic acid) was added to the protein at four molar equivalents per cysteine conjugation site. The reductant was thoroughly mixed into each protein sample and placed at 37 °C on a nutator for 4 h or until cysteine decapping went to completion, as assessed by LCMS. Following decapping, excess reductant was removed using a Cytiva G-25 desalting column. The decapped protein was then reacted with four equivalents of the at 24 °C for 2–4 h or until the reaction went to completion. Excess linker–payload was then removed using a Cytiva G-25 desalting column, followed by buffer exchange using a 3K MWCO spin column (MilliporeSigma, Burlington, MA, USA).

### 2.4. Liquid Chromatography–Mass Spectrometry (LCMS)

LCMS analysis was performed on an Agilent TOF 6230 (G6230B, SG19060102; Agilent, Santa Clara, CA, USA) equipped with a BioResolve RP mAb Polyphenyl Column (450 Å, 2.7 µm, 2.1 mm × 150 mm (Waters Part No.: 186008946, Milford, CA, USA)) and operated at a flow rate of 0.5 mL/min in 15% acetonitrile with 0.1% TFA. Data were processed using Agilent’s Qualitative Analysis software (version B0.07.00).

### 2.5. Conjugation Stability Testing

Following sample conjugation, an initial LCMS trace was acquired to confirm full protein modification. Protein conjugates were then mixed with the mother liquor used for crystallization studies and incubated at room temperature for twenty days.

Protein conjugate binding slurry (20 μL of CaptureA for Fabs or PrismA (Cytiva) beads for Fcs) was added to centrifugal 3K MWCO spin columns (MilliporeSigma) and centrifuged to remove supernatant. The beads were then washed four times with 200 μL Binding Buffer (20 mM HEPES pH 7.5, 150 mM NaCl) and centrifuged between washes to remove the supernatant. Protein conjugates in their crystallization buffer were added to the beads and allowed to bind for 15 min on a nutator. Samples were centrifuged and the flowthrough was discarded, after which the samples were washed four times with 200 μL Binding Buffer. Samples were eluted with 100 mM Glycine pH 3.5 in three separate elution spins (10, 20, and 10 μL). Eluted samples were then analyzed by LCMS. Data was processed using Agilent Quantitative Analysis software (version B0.07.00).

### 2.6. Crystallization

Fab A172C and Fc S375C/Q362C samples were buffer exchanged into crystallization buffer consisting of 20 mM HEPES pH 7.5, 100 mM NaCl and concentrated to 10 mg/mL for the Fab A172C sample and 35 mg/mL for the Fc S375C/Q362C sample. Crystallization was performed by sitting-drop vapor diffusion using a 1:1 ratio of protein to reservoir solution at 20 °C. Microseeding was employed during optimization to promote crystal growth. Crystals of the Fab A172C mutant belonging to the space group C 1 2 1 were obtained using a reservoir solution containing 0.09 M HEPES pH 7.5, 0.0045 M CdCl_2_, 0.0045 M CoCl_2_, 0.0045 M MgCl_2_, 0.0045 M NiCl_2_, 10.8% (*w*/*v*) PEG 3350, and 4% (*v*/*v*) 1,3-butanediol. Crystals were harvested 22 days after setup using 25% glycerol as a cryo-protectant. Crystals for the Fc S375C Q362C mutant, which crystallized in space group P 2_1_ 2_1_ 2_1_, grew using a reservoir solution containing 0.1 M MES pH 6.5, 5% (*v*/*v*) MPD, and 15% (*w*/*v*) PEG 6K. Crystals were harvested 12 days after setup, also using 25% glycerol as a cryo-protectant.

### 2.7. Crystallography Data Collection and Structure Determination

Fab A172C X-ray diffraction data were collected at Brookhaven National Laboratory’s National Synchrotron Light Source II on beamline 17-ID-2 (AMX) on an Eiger 16M detector (Dectris, Baden, Switzerland).

Fc S375C/Q362C X-ray diffraction data were collected through the Industrial Macromolecular Crystallography Association (IMCA) at the Canadian Light Source beamline 08B1-1 on a Pilatus 3 S 6M detector (Dectris).

All data were processed using autoPROC (version 1.1.7) and elliptically truncated using STARANISO (version 2.4.16) [29,30]. All structures were solved by molecular replacement using Phaser within the Phenix software (version 1.19.2_4158) with PDB search models 1N8Z (Fab only) and 3AVE for Fab and Fc, respectively [31,32]. The structures were manually rebuilt in Coot (version 0.9.8.94) and refined using Phenix [33]. Data processing and refinement statistics can be found summarized in Table 2.

### 2.8. Hydrophobic Interaction Chromatography (HIC) of Antibodies and Antibody–Drug Conjugates

HIC analysis of antibodies and antibody–drug conjugates was performed on an Agilent HPLC 1260 system equipped with a diode array and a fluorescence detector. Separation was achieved using a TOSOH TSKgel Butyl-NPR (4.6 mm × 10 cm) column (TOSOH, Tokyo, Japan) using a gradient from 100% 1.5 M ammonium sulfate in 50 mM sodium phosphate (pH 7.0) to 100% 50 mM sodium phosphate (pH 7.0). Five μL of antibodies or 15 μL of ADCs at a concentration of 1 mg/mL was loaded and eluted with a flow rate = 0.5 mL/min; gradient elution = 40 min linear gradient of 30–80% 50 mM sodium phosphate (pH 7.0). The eluent was monitored by excitation at 280 nm and emission detection at 348 nm.

## 3. Results

### 3.1. Molecular Dynamics-Guided Selection of Site-Specific Cysteine Mutants for Targeted ADC Exploration

**Figure 1:**
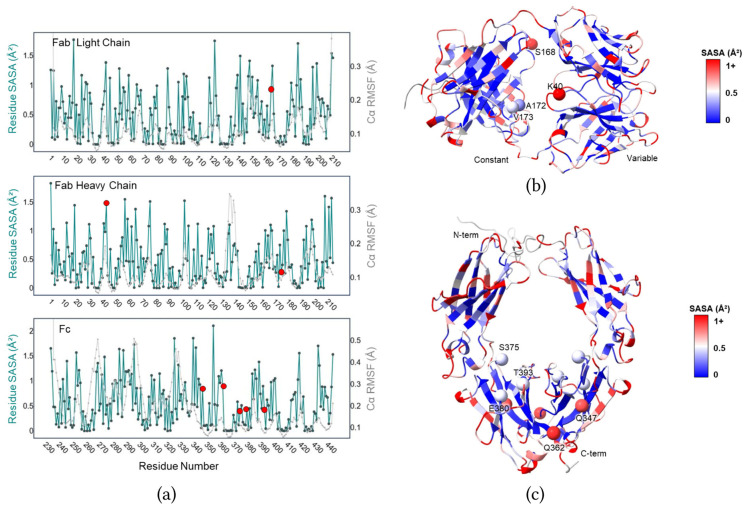
Selected cysteine mutants based on SASA and Cα RMSF values. (**a**) Residue-by-residue plots of trastuzumab amino acid SASA and RMSF of Cα atoms, calculated following molecular dynamics simulations. Residues selected for site-selective mutation are denoted with red circles. (**b**,**c**) Cartoon representations of trastuzumab Fab (**b**) and Fc (**c**) structures (Trastuzumab homology model) colored according to residue SASA values. Residues investigated in the present study are labeled and their Cα atoms shown as sphere representations. Fc spheres are illustrated in both monomers.

In selecting trastuzumab cysteine mutations for site-selective linker payload conjugation, we hypothesized that conjugation within or near large surface pockets would shield the large and intrinsically flexible ADC linker–payload. To identify antibody residues of interest, molecular dynamics (MD) simulations were run on both the Fab (PDB 5GGS, 100 ns) and Fc (PDB 3AVE, 200 ns) fragments of trastuzumab. MD traces were then analyzed for average residue-level solvent-accessible surface area (SASA) and Cα root mean square fluctuation (RMSF) (Figure 1a–c). While near-zero SASA scores suggest potential labeling accessibility issues, a low to medium SASA score may suggest an increase in antibody–payload shielding, potentially providing an environment that favors antibody–linker interactions. Similarly, low Cα RMSF suggests increased residue rigidity, which may allow for more stable antibody–linker interactions. Based on our analysis, we selected residues with a range of SASA and Cα RMSF values around the main Fab pocket and the main Fc pocket (excluding the CH2 glycan region) for cysteine mutagenesis and structural studies. These residues include Fab light chain mutant S168 and heavy chain mutants K40C, A172C, and V173C, as well as the Fc mutants Q347C, Q362C, S375C, E380C and T393C.

### 3.2. Selected Cysteine Mutants in Trastuzumab Fragments Are Homogenously Pure and Monodisperse Post Conjugation

**Figure 2:**
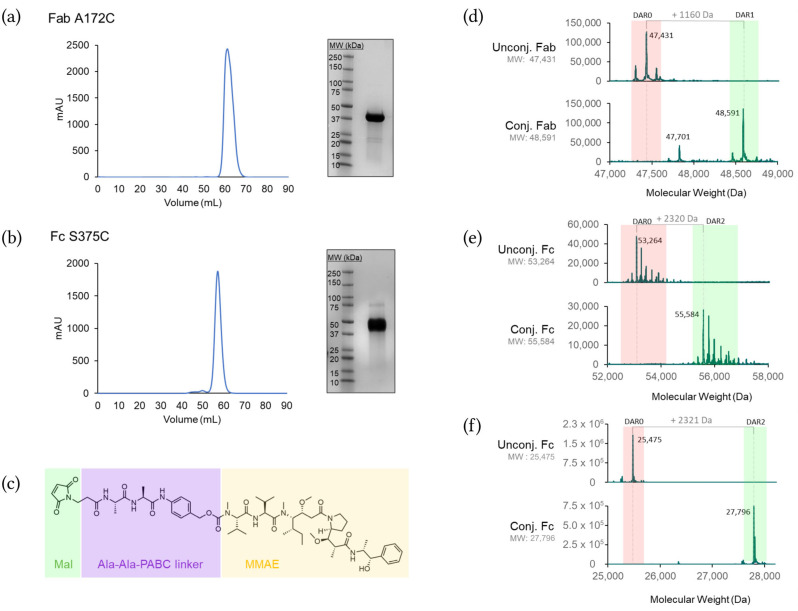
Purification and linker–payload conjugation of Fab and Fc site-specific cysteine mutants. (**a**,**b**) (**left***)* Superdex 75 size exclusion chromatograms for Fab and Fc site-specific cysteine mutants. (**right**) SDS-PAGE gel analysis of final protein samples used for protein crystallography. (**c**) Chemical structure of the ADC linker–payload used herein. The molecule consists of a maleimide (Mal) handle (green), an Ala-Ala-PABC linker (purple) and an MMAE payload (yellow). (**d**–**f**) LC/MS spectra for Fab (A172C, (**d**)), hinged Fc (S375C, (**e**)) and non-hinged Fc (Q323C/S375C; (**f**)) site-specific cysteine mutants before and after conjugation of the linker payload in DAR1, DAR2, or DAR4 ratios, respectively.

To facilitate crystallization, site-specific cysteine mutants of the trastuzumab monoclonal antibody (mAb) were expressed from ExpiCHO cells as either Fab or Fc truncations. These Fab and Fc mutants were purified using MabSelect resin for affinity capture, followed by size-exclusion chromatography (Figure 2a,b). For the Fc mutants, two constructs were used: one with a minimal hinge region removed (hinge-free Fc) and one retaining the hinge (hinged Fc).

**Figure 3:**
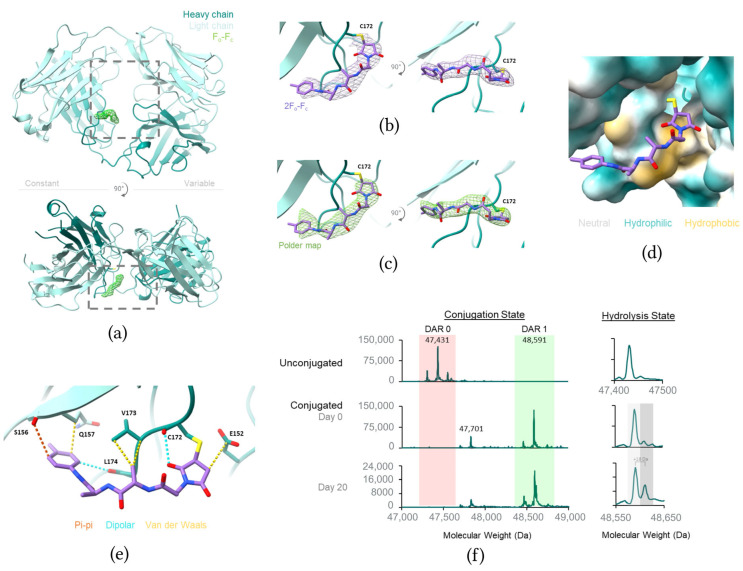
Structural analysis of ADC linker interactions at the A172C Fab conjugation site. (**a**) Crystal structure of the trastuzumab Fab A172C mutant conjugate with unassigned F_o_-F_c_ omit density contoured to 2.5 *σ* (green mesh). Fab pocket is indicated by gray dashed box. (**b**) Close-up view of the ADC linker (purple stick representation) conjugated to C172 of the trastuzumab Fab with 2F_o_-F_c_ density contoured to 1.0 *σ* (purple mesh) or (**c**) a polder map contoured to 2.5 *σ* (green mesh). (**d**) Surface representation of the ADC linker binding site. The surface is colored according to hydrophobicity (hydrophilic in teal, neutral in white, hydrophobic in yellow mustard). (**e**) Close-up view of the ADC linker binding site with pi–pi interactions (orange), dipolar interactions (teal), and van der Waals interactions (yellow) shown in dashed lines. (**f**) (**left**) LCMS deconvolution spectra showing the stability of the A172C conjugate over time in crystallization buffer and (**right**) the hydrolysis state of the conjugated maleimide.

**Figure 4:**
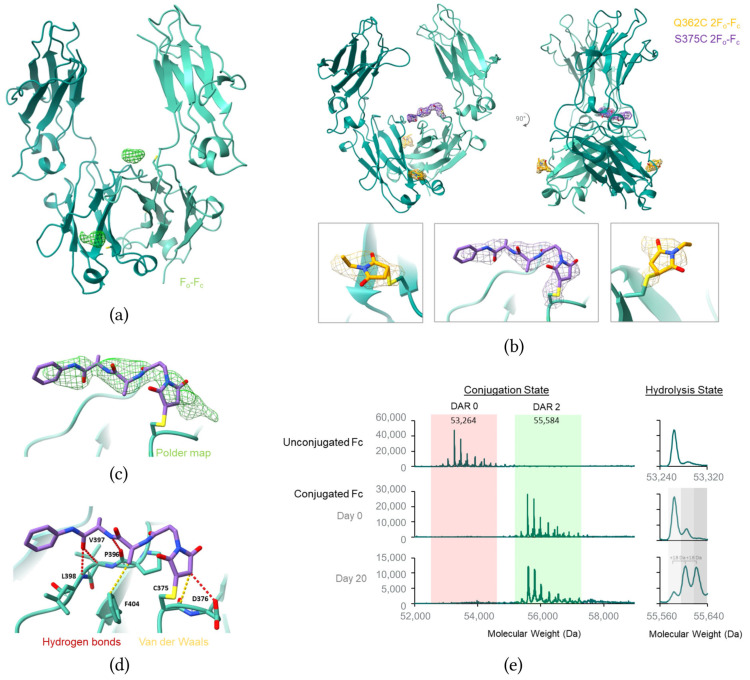
Structural analysis of ADC linker interactions at the S375C Fc conjugation site. (**a**) Crystal structure of the trastuzumab Fc S375C/Q362C mutant with unassigned F_o_-F_c_ omit density contoured to 2.5 *σ* (green mesh). (**b**) (**top**) Front and side views of conjugation sites Q362C/S375C of the trastuzumab Fc with 2F_o_-F_c_ map contoured to 1.0 *σ* (purple and orange mesh). (**bottom**) Close-up views of the ADC linkers conjugated to the Q362C and S375C mutants of trastuzumab (orange and purple stick representation, respectively) with 2F_o_-F_c_ maps contoured to 1.0 *σ* (orange and purple mesh, respectively). (**c**) Close-up view of ADC linker conjugated to S375C of the trastuzumab Fc with polder map contoured to 2.5 *σ* (green mesh). (**d**) Close-up view of the S375C ADC linker binding site with key interactions shown in dashed lines. (**e**) LCMS deconvolution spectra showing the stability of the S375C conjugate over time in crystallization buffer (**left**) and the hydrolysis state of the conjugated maleimide (**right**).

To selectively reduce engineered cysteines without disrupting native interchain disulfide bonds, we used 4,4′-(Phenylphosphinidene)bis-benzenesulfonic acid, a phosphine reagent that preferentially targets hydrophobic sites [34]. The Fab and Fc fragments were subsequently conjugated with an Ala-Ala-PABC linker attached to an MMAE payload (Figure 2c). LCMS analysis confirmed that labeling went to completion (Figure 2d–f), with some mutants undergoing maleimide hydrolysis (Supplementary File Figure S4a, Figure 3f and Figure 4e), a modification known to enhance the stability of maleimide drug conjugates [22].

### 3.3. The Fab A172C Conjugate Linker Specifically Interacts with the Trastuzumab Fab

Fab A172C conjugate crystals grew upon seeding with apo Fab crystals and diffracted to a resolution of 2.4 Å (Table 1; crystallographic B-factors shown in Supplementary File Figure S1a). Analysis of the Fab pocket between the variable and constant regions revealed clear F_o_-F_c_ electron density adjacent to the sulfhydryl group at the A172C conjugation site (Figure 3a). While the linker portion of the linker–payload was well resolved and modeled (Figure 3b,c), only weak discontinuous payload density was present near the linker, suggesting that the payload itself makes few, if any, specific interactions with the Fab (Supplementary File Figure S2a,b).

The Ala-Ala-PABC linker conjugated to A172C binds in a hydrophobic region in the Fab pocket, burying 326 Å^2^ of solvent-accessible surface area (Figure 3d). The maleimide forms van der Waals contacts with E152 as well as dipole–dipole interactions with the backbone carbonyl of C172. Weak van der Waals interactions with the stacked Y180 may contribute to stabilizing the maleimide–Fab interface. The first alanine Cβ of the linker interacts via van der Waals interactions with both the Cα and sidechain of V173. The benzyl moiety engages in multiple interactions, including a van der Waals interaction with the Cβ of E157, a dipolar interaction with the backbone carbonyl of L174 and pi–pi interaction with the backbone carbonyl of S159. Hydrophobic residues such as V173, L174, Y180 and Y149 shield roughly half of the linker’s solvent accessibility (Figure 3e). This detailed visualization of the Fab–linker interaction provides a foundation for the structure-based design of next-generation linker–payloads that may bind more tightly at this site, potentially improving the systemic stability of ADCs. The presence of the payload in the crystals was confirmed by LCMS following a 20-day incubation in the crystallization solution at room temperature (Figure 3f).

Despite extensive crystal screening efforts, crystals were not obtained for trastuzumab Fab conjugates at sites K40C, S168C and V173C even with seeding, potentially due to flexibility at the conjugation sites or interference from the linker–payload near crystal contacts in this crystal form (Supplementary File Figure S2c,d).

### 3.4. Fc S375C/Q362C Conjugate Structure Suggests the Central Fc Pocket Protects the Conjugated Linker

Crystals of the Fc S375C/Q362C DAR4 conjugate diffracted to a resolution of 2.5 Å. Analysis of crystallographic B factors revealed lower values in the CH3 domains, indicating reduced thermal motion, while higher B factors were observed near the Fc-Fab hinge region of the CH2 domain (Table 1, Supplementary File Figure S1b). To optimize structural studies, both hinge-containing and hinge-free Fc constructs were assessed; the hinge-free construct was ultimately selected because it consistently produced higher-quality crystals and enabled data collection at improved resolution. Distinct positive Fo-Fc electron density was detected at the engineered cysteine residues Q362C and S375C, though the signal intensity varied between the two sites (Figure 4a). At the more solvent-exposed Q362C conjugation site, the 2F_o_-F_c_ density was sufficient only to model the conjugated maleimide moiety of the linker–payload (Figure 4b). In contrast, the 2F_o_-F_c_ density at the S375C site, located in the central pocket of the Fc dimer, was sufficient to model the majority of the Ala-Ala-PABC linker in one Fc monomer (Figure 4b). Notably, the second monomer showed limited linker density, likely reflecting increased linker–payload flexibility and indicating weaker interactions at this site (Supplementary File Figure S3a–c). As observed for the Fab A172C structure, no clear electron density was observed for the ADC payload itself, despite LCMS confirmation of its presence (Figure 4e).

The difference in electron density strength between Q362C and S375C conjugation sites is likely attributable to the solvent protection provided by the Fc pocket (Figure 1a) and the specific interactions available to the linker at each site (Figure 4d). While the linker density at one of the S375C conjugation sites is stronger compared to the density at Q362C, the 2Fo-Fc and polder maps at the S375C site reveal some ambiguity, suggesting heterogeneous linker conformations (Figure 4b,c). The data at this site is best modeled by the conjugated, non-hydrolyzed maleimide linker depicted in Figure 4, although both hydrolyzed forms were also considered (Supplementary File Figure S4a–e).

Detailed examination of the structure reveals two canonical hydrogen bonds: one between the linker NH and the backbone carbonyl of P296 and another between the linker carbamate and the main chain amide of L398 (Figure 4d). Additionally, the terminal linker amide carbonyl forms a non-canonical hydrogen bond with the Cα of V397. The maleimide group forms a hydrogen bond with the carboxyl side chain of D376 and a van der Waals interaction with the D376 backbone, which may help stabilize the maleimide in this non-hydrolyzed form. Several nonpolar residues surrounding the S375C site including P396, L398, V397 and F404 contribute to a buried surface area of 244 Å^2^, including a specific van der Waals interaction between F404 and the first alanine methyl group on the linker.

Various other Fc conjugate mutants (S347C, E380C, E382C, T393C) were also tested for crystallization. Crystals were successfully obtained only for S375C and Q362C/S375C mutants.

### 3.5. Maleimide Hydrolysis Likely Contributes to the Observed Heterogeneity at the S375C Site

Consistent with the ambiguous linker density at the S375C site, LCMS analysis of the conjugated Fc reveals a mass shift of 18 or 36 Da, indicating that maleimide hydrolysis occurs at one or both S375C sites of the Fc (Figure 4e). Maleimide hydrolysis involves an opening of the maleimide ring, enhancing conjugation stability by preventing the retro-Michael reaction that would release the conjugate altogether (Supplementary File Figure S4a). This hydrolysis suggests that the density observed in the S375C structure likely represents a mixture of hydrolyzed and non-hydrolyzed maleimide linker–payloads conjugated to C375. While structural data are best modeled by the non-hydrolyzed maleimide conjugate, the conformational state that is modeled represents one state in a dynamic mixture consisting of both hydrolyzed and non-hydrolyzed forms (Supplementary File Figure S4b–e). Further studies may focus on optimizing interactions with hydrolyzed conjugates to further improve the systemic stability of ADCs.

### 3.6. Stronger Antibody–Linker Interactions Correlate with Lower ADC Hydrophobicity

The antibody–linker interactions observed in our structures suggest that antibody pockets shield the relatively hydrophobic ADC linker and payload, potentially increasing ADC solubility. To test this hypothesis, we compared the hydrophobicity of two pocket ADC mutants, A172C (Fab) and S375C (Fc), with the more exposed K40C (Fab) mutant (Supplementary File Figure S5a). Crystallography studies suggested the strongest antibody–linker interactions in the Fab A172C mutant, followed by the Fc S375C interactions, while no crystals were obtained for the more exposed Fab K40C mutant. Hydrophobic Interaction Chromatography (HIC) profiles of the full IgG mutant ADCs followed a similar trend: the exposed K40C ADC exhibited the highest hydrophobicity, followed by the Fc pocket S375C ADC and finally the most shielded A172C ADC. These findings support the notion that engineering linker–payloads to interact closely with antibody pockets may contribute to lower ADC hydrophobicity.

## 4. Discussion

Recent advances in site-specific conjugation strategies have significantly improved homogeneity of antibody–drug conjugates by enabling precise attachment of linker–payload. Additionally, the strategy has improved thermostability through linker–payload distancing [15] and increased conjugation stability via higher maleimide hydrolysis rates [23]. These advances have largely been driven by computational approaches [17,34], high-throughput mutational scanning [35], and mutagenesis based on structural insights [36]. Building on this foundation, our study demonstrates the added value of structural biology for directly visualizing and potentially improving site-specific linker–antibody interactions, thereby expanding the toolkit for ADC development.

Using trastuzumab conjugated with an Ala-Ala-PABC-MMAE linker–payload as a model system, we have provided structural insights into specific interactions formed when conjugation is carried out at two distinct sites: A172C on the Fab and S375C on the Fc domain. Both sites are situated near sizeable pockets that shield and stabilize fortuitous linker–antibody interactions, allowing for high-resolution structure determination. Although our studies show no evidence of payload binding to trastuzumab, these initial ADC structures lay the groundwork for structure-based drug design aimed at optimizing linkers that improve the interactions at each site, potentially leading to ADCs with enhanced systemic stabilities. The present study suggests that molecular dynamics and HIC may be effective screening tools for future studies, guiding site-specific cysteine mutagenesis and enabling comparative structural studies of a diverse set of ADCs.

At the Fab A172C site, the linker binds within a pocket formed between the Fab’s constant and variable regions. This pocket is rich in hydrophobic residues and has previously been explored as a potential site of hydrophobic shielding through ADC design [34]. Our findings confirm that the Ala-Ala-PABC linker engages closely with this pocket, effectively shielding approximately half of the linker while leaving the remainder of the linker exposed to solvent. Future work will explore how the Ala-Ala-PABC linker used in this study might be further engineered to enhance these interactions. Additionally, although we examined only one linker–payload in this study, future studies could explore different linker–payloads with distinct characteristics to determine the effects of these changes on linker interactions within this Fab pocket.

While the electron density from our Fab dataset allowed for clear linker modeling, the linker density from our Fc dataset was far more ambiguous, and it is therefore critical to note that the resulting model represents one possible state in a complex and dynamic mixture. Several factors likely contributed to this complexity. First, LCMS analysis revealed that the Fc S375C site undergoes a higher rate of maleimide hydrolysis compared to the Fab A172C site. Maleimide hydrolysis irreversibly stabilizes the conjugation but also introduces structural heterogeneity by generating two products with the cysteine conjugated to either of two adjacent carbons (Supplementary File Figure S4) [23]. Second, maleimide hydrolysis leads to ring opening of the conjugation handle, increasing the linker’s rotational degrees of freedom at the conjugation site. Finally, the generally weaker or less-specific interactions between the linker and the Fc pocket may result in lower occupancy of the bound conformation and therefore weaker electron density. Future structural studies would benefit from using fully hydrolyzed maleimide-based ADCs or more homogenous conjugation chemistries to reduce heterogeneity.

This work investigated a single antibody scaffold (trastuzumab) conjugated to one type of linker–payload (Ala-Ala-PABC-MMAE). While the conjugation sites and the antibody–linker interactions identified here may not be directly generalizable to other antibodies or chemically diverse linker–payloads, this study provides a proof-of-concept and an experimental framework for using structural biology to identify analogous conjugation sites in other ADCs of interest. Although we were unable to resolve the MMAE payload at any of the conjugation sites investigated, this may be achievable for other ADCs. Continued efforts are essential for expanding this method’s applicability. Strategies such as allowing complete maleimide hydrolysis where possible, improving conjugation methods, and computationally designing linkers based on potential antibody interactions could accelerate ADC structure-based design in the future. Importantly, it remains unclear whether these findings will be translatable to clinical settings. Future work should be aimed at using the site-specific conjugates described here—as well as their derivatives obtained through structure-based design—to assess the impact of increased linker shielding on various aspects of ADC pharmacology, including serum stability, efficacy, and toxicity.

In summary, our findings highlight the potential of structural biology in characterizing the IgG–linker interactions of ADCs and guiding linker modifications to strengthen interactions that may shield linkers and/or linker–payloads. Such optimized linkers could reduce ADC aggregation, prevent premature payload release, and improve overall ADC pharmacokinetics, thereby advancing the therapeutic efficacy and safety of next-generation ADCs.

## Figures and Tables

**Table 1:** Protein constructs used in the current study.

Construct Name	Amino Acid Sequence
Fc Q362C S375C	LLGGPSVFLFPPKPKDTLMISRTPEVTCVVVDVSHE DPEVKFNWYVDGVEVHNAKTKPREEQYNSTYRV VSVLTVLHQDWLNGKEYKCKVSNKALPAPIEKTI SKAKGQPREPQVYTLPPSRDELTKNCVSLTCLVKG FYPCDIAVEWESNGQPENNYKTTPPVLDSDGSFFL YSKLTVDKSRWQQGNVFSCSVMHEALHNHYTQK SLSLSPGK
Fc hinge S375C	MGWSCIILFLVATATGVHSTHTCPPCPAPELLGGP SVFLFPPKPKDTLMISRTPEVTCVVVDVSHEDPEVK FNWYVDGVEVHNAKTKPREEQYNSTYRVVSVLT VLHQDWLNGKEYKCKVSNKALPAPIEKTISKAKG QPREPQVYTLPPSRDELTKNQVSLTCLVKGFYPCD IAVEWESNGQPENNYKTTPPVLDSDGSFFLYSKLTV DKSRWQQGNVFSCSVMHEALHNHYTQKSLSLSPGK
Fab Light Chain	MGWSCIILFLVATATGVHSDIQMTQSPSSLSASVG DRVTITCRASQDVNTAVAWYQQKPGKAPKLLIYS ASFLYSGVPSRFSGSRSGTDFTLTISSLQPEDFATYY CQQHYTTPPTFGQGTKVEIKRTVAAPSVFIFPPSD EQLKSGTASVVCLLNNFYPREAKVQWKVDNALQ SGNSQESVTEQDSKDSTYSLSSTLTLSKADYEKHK VYACEVTHQGLSSPVTKSFNRGEC
Fab A172C Heavy Chain	MGWSCIILFLVATATGVHSEVQLVESGGGLVQPG GSLRLSCAASGFNIKDTYIHWVRQAPGKGLEWV ARIYPTNGYTRYADSVKGRFTISADTSKNTAYLQ MNSLRAEDTAVYYCSRWGGDGFYAMDYWGQG TLVTVSSASTKGPSVFPLAPSSKSTSGGTAALGCLV KDYFPEPVTVSWNSGALTSGVHTFPCVLQSSGLY SLSSVVTVPSSSLGTQTYICNVNHKPSNTKVDKKV EPKSCDK

**Table 2:** Data collection and refinement statistics.

	Trastuzumab Fab A172C Conjugate	Trastuzumab Fc Q362C/S375C Conjugate
Wavelength	0.97934 Å	1.18053 Å
Resolution range (ellipsoidal)	64.29–2.42 (2.58–2.42)	69.15–2.69 (2.73–2.68)
Resolution range (isotropic)	64.29–2.65 (2.70–2.5)	69.15–2.46 (2.66–2.46)
Space group	C 1 2 1	P 21 21 21
Unit cell	128.90 64.94 83.81 90.0 129.91 90.0	49.52 79.69 138.29 90.0 90.0 90.0
Total reflections	103,793	195,096
Unique reflections	14,858	15,154
Multiplicity	7.0	12.9
Completeness anisotropic (%)	87.4	93.4
Completeness isotropic (%)	94.4	99.9
Mean I/sigma(I)	11.5	15.8
R_merge_ ^a^	0.084 (1.925)	0.117 (2.118)
R_meas_ ^b^	0.092 (2.080)	0.122 (2.217)
R_pim_ ^c^	0.035 (0.780)	0.034 (0.645)
CC_1/2_	0.983 (0.455)	0.999 (0.464)
Reflections used in refinement	14,831	15,162
R-work	0.241	0.248
R-free	0.290	0.272
Number of non-hydrogen atoms	3221	3545
macromolecules	3190	3287
ligands	31	246
solvent	0	12
Protein residues	421	414
RMS (bonds)	0.003	0.002
RMS (angles)	0.67	0.50
Ramachandran favored (%)	96.37	99.76
Ramachandran allowed (%)	3.63	0.24
Ramachandran outliers (%)	0.00	0.00
Rotamer outliers (%)	1.68	0.53
Clashscore	10.78	6.11
Average B-factor	70.62	68.68
macromolecules	70.52	66.35
ligands	80.45	100.68
solvent		51.90

Statistics for the highest-resolution shell are shown in parentheses. ^a^ R_merge_ = $\sum_{hkl}\sum_{i}\left|I_{i}\left(hkl\right)-\bar{I_{i}\left(hkl\right)}\right|/\sum_{hkl}\sum_{i}I_{i}(hkl)$, ^b^ R_meas_ *=* $\sum_{hkl}\sqrt{\frac{N}{N-1}}\sum_{l}\left|I_{l}\left(hkl\right)-\bar{I_{i}\left(hkl\right)}\right|/\sum_{hkl}\sum_{i}I_{i}(hkl)$, ^c^ R_pim_ *=* $\sum_{hkl}\sqrt{\frac{1}{N-1}}\sum_{l}\left|I_{l}\left(hkl\right)-\bar{I_{i}\left(hkl\right)}\right|/\sum_{hkl}\sum_{l}I_{i}(hkl)$.

## Data Availability

The final coordinates of the trastuzumab Fab A172C conjugate and the trastuzumab Fc Q362C/S375C conjugate have been deposited in the PDB with accession codes 9YZZ and 9Z0F, respectively. Source data are provided with this paper. All other data generated in this study are available in the Supplementary File as well as from the authors upon request.

## Supplementary Materials

The following supporting information can be downloaded at: https://www.mdpi.com/article/10.3390/pharmaceutics17121568/s1, Figure S1: Fab and Fc conjugate structures colored according to crystallographic B-factors.; Figure S2: Trastuzumab Fab A172C crystal packing interactions.; Figure S3: S375C mutations in the main Fc dimer pocket have differing electron densities.; Figure S4: Alternative models of the S375C conjugated linker given maleimide hydrolysis.; Figure S5: Hydrophobic interaction chromatography elution profiles for site-specific IgG conjugates.

## Abbreviations

The following abbreviations are used in this manuscript:

ADC	Antibody Drug Conjugate
CH2/CH3	antibody Constant Heavy domains
DAR	Drug Antibody Ratio
HIC	Hydrophobic Interaction Chromatography
IMCA	Industrial Macromolecular Crystallography Association
Fab	Fragment antigen-binding
Fc	Fragment crystallizable
HPLC	High-Performance Liquid Chromatography
LCMS	Liquid Chromatography–Mass Spectrometry
Mal	Maleimide
MMAE	Monomethyl auristatin E
mAb	monoclonal Antibody
PABC	P-AminoBenzyloxyCarbonyl
RMS	Root Mean Square
RMSF	Root Mean Square Fluctuation
SASA	Solvent-Accessible Surface Area
VH/VL	Variable Heavy/Variable Light chains
