# Supplementary material for: Structural Characterization of Linker Shielding in ADC Site-Specific Conjugates
Source: Pharmaceutics. 2025 Dec 5;17(12):1568. doi: 10.3390/pharmaceutics17121568 (PMC12736158; doi:10.3390/pharmaceutics17121568)
Supplement: Supplementary file 1 [file pharmaceutics-17-01568-s001.zip › pharmaceutics-3990879-supplementary.pdf]

Structural characterization of linker shielding in ADC site-specific conjugates

Maru Jaime-Garza [1], Andrew Waight [2], Manish Hudlikar [1], Michael J. Eddins [3], Elnaz S. Rasti [2], Jan Paulo T. Zaragoza [2], Laurence Fayadat-Dilman [2], Jill E. Chrencik [1], Sandra B. Gabelli [3], Yun-Ting Chen [4], Cameron L. Noland [1,*]

Supplementary Data

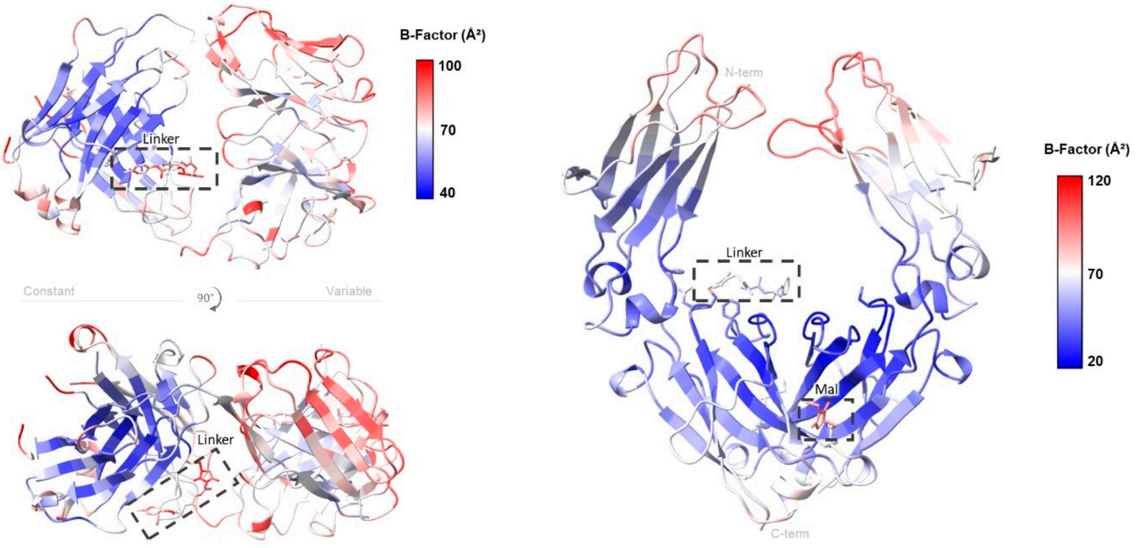

(a) (b)

Figure S1. Fab and Fc conjugate structures colored according to crystallographic B-factors. a Trastuzumab Fab A172C conjugate structure shown in cartoon representation and colored according to the crystallographic b factors or Thermal/Brownian motion where blue is lower than 70 Å^2 and red is higher than 70 Å^2. Linker highlighted by dashed box. **b** Trastuzumab Fc S375C/Q362C conjugate structure shown in cartoon representation and colored according to the crystallographic b factors or Thermal/Brownian motion where blue is lower than 70 Å^2 and red is higher than 70 Å^2. Maleimide and linker highlighted by dashed box.

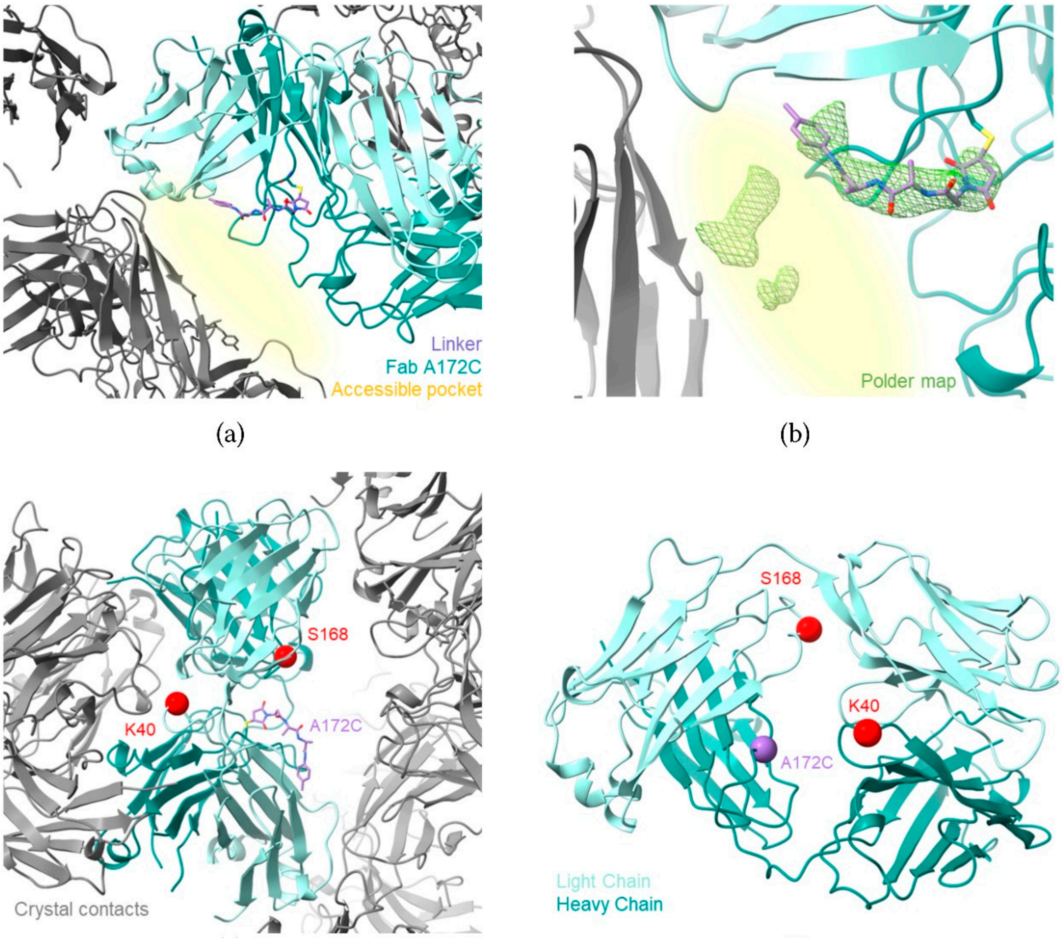

(a) (b) (c) (d)

Figure S2. Trastuzumab Fab A172C crystal packing interactions. a Cartoon representation of Fab A172C crystal packing with the molecule within the asymmetric unit shown in teal cartoon representation and symmetry mates is shown in grey cartoon representation. A solvent channel adjacent to the conjugated ADC linker (purple stick representation) is highlighted with a yellow oval. **b** Polder map (green mesh, 2.5 σ) shows unmodeled density in the solvent channel (highlighted by yellow oval) directly adjacent to the conjugated ADC linker (purple stick representation). **c** Trastuzumab A172C Fab conjugate crystal structure with symmetry mates shown in grey cartoon representation and the K40 and S168 Cα atoms shown as red spheres. **d** The K40 and S168 conjugation positions (Cα atoms shown as red spheres) are located in loops, while the A172 site (Cα atom shown as purple sphere) is located between the Fab's variable and constant regions.

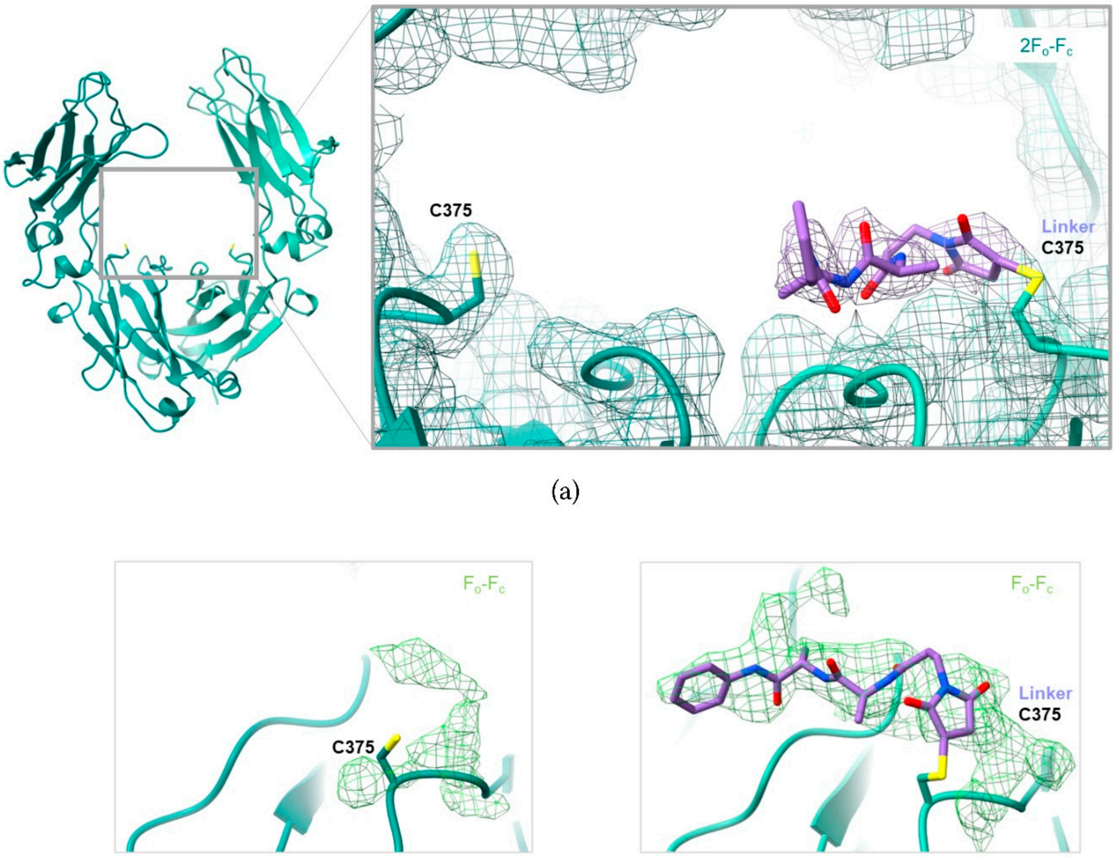

(a)

(b)

(c)

Figure S3. S375C mutations in the main Fc dimer pocket have differing electron densities. **a** Close up of Fc pocket with 2Fo-Fc electron density contoured to 1 σ. The linker for one Fc monomer is shown in purple stick representation. **b,c** Fo-Fc difference maps at C375 of each Fc monomer (green mesh, 1.5 σ). The modeled linker is shown in purple stick representation in **c**.

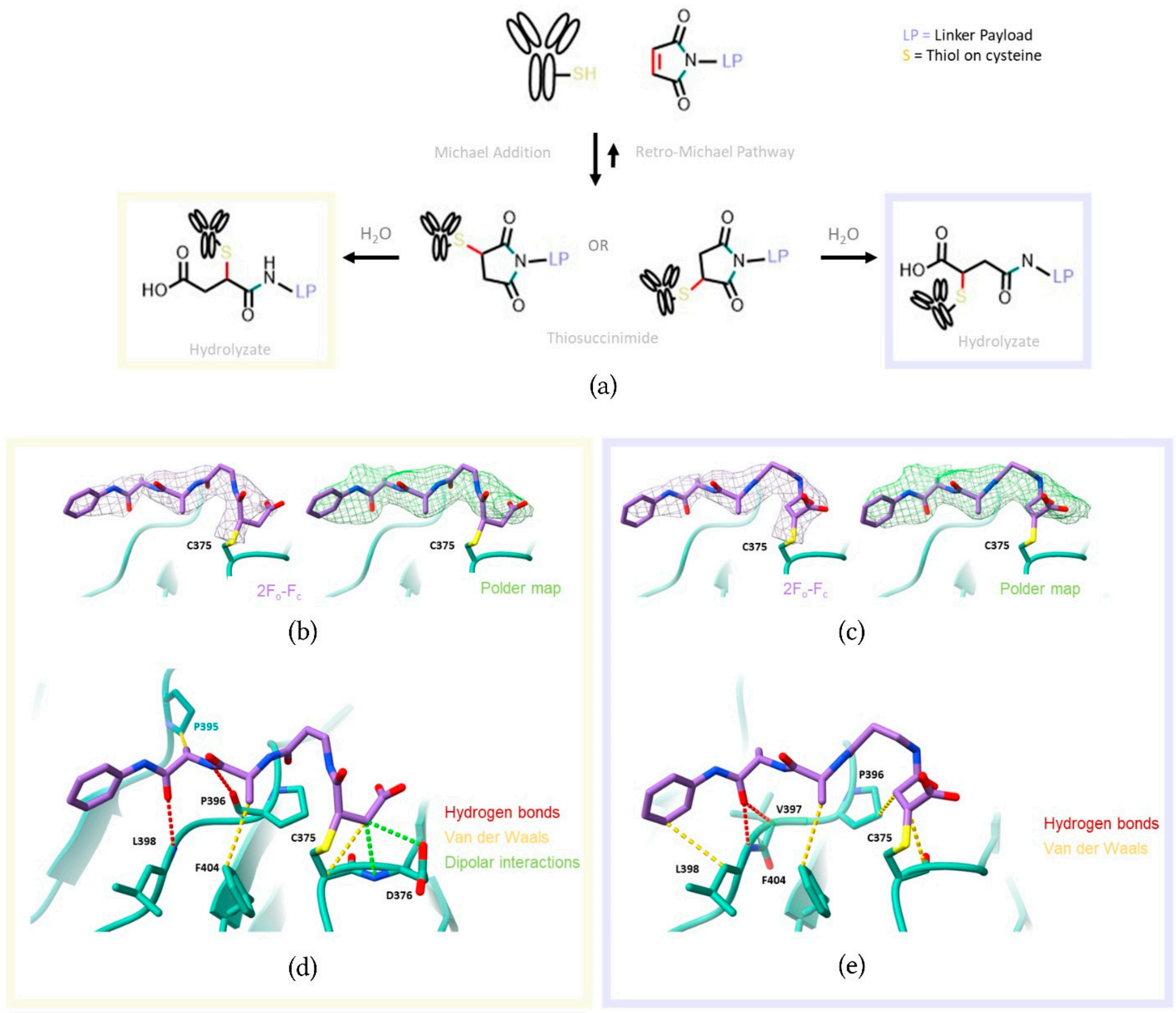

Figure S4. Alternative models of the S375C conjugated linker given maleimide hydrolysis. a Schematic of reversible thiol-maleimide conjugation reactions and irreversible hydrolysis reaction. Conjugation occurs at either of two adjacent maleimide carbons. Irreversible hydrolysis opens the maleimide ring with the linker conjugated at either of two adjacent carbons. **b,c** 2 Fo-Fc (purple mesh, 1 σ) and Polder maps (green mesh, 2.5 σ) with each possible hydrolyzed forms of the linker modeled in purple sticks. **d,e** Close-up view of the linker binding site with key interactions for each hydrolyzed model shown in dashed lines.

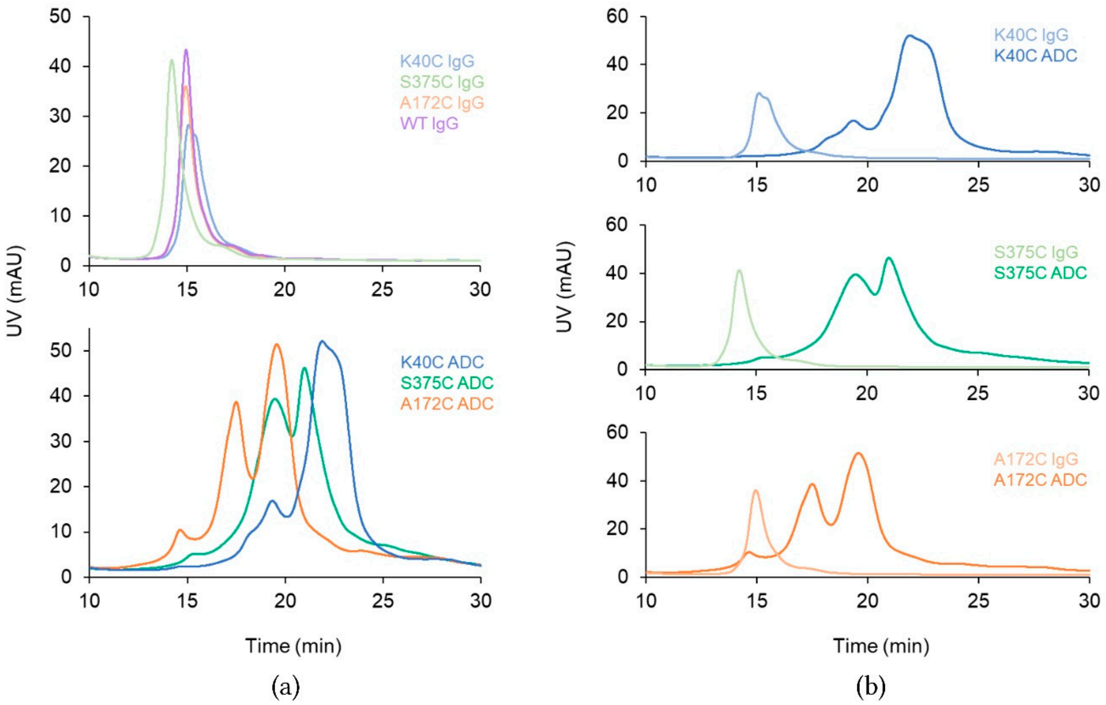

Figure S5. Hydrophobic interaction chromatography elution profiles for site-specific IgG conjugates. a Overlays of unconjugated cysteine IgG mutant (top) and cysteine-mutant ADC (below) HIC elution profiles. Multiple peaks in ADC traces show DAR1 and DAR2 populations. **b** IgG mutants overlayed with corresponding ADC HIC elution profiles.
